# The Molecular Landscape of CASTLE: A Rare Thymus-like Head and Neck Cancer

**DOI:** 10.3390/ijms27083501

**Published:** 2026-04-14

**Authors:** William C. Cho, Allen C. S. Yu, Wah Cheuk, Aldrin K. Y. Yim, James C. H. Chow, John K. C. Chan, Ka M. Cheung

**Affiliations:** 1 Department of Clinical Oncology, Queen Elizabeth Hospital, Hong Kong, China; 2 Codex Genetics Ltd., Hong Kong, China; 3 Department of Pathology, Queen Elizabeth Hospital, Hong Kong, China

**Keywords:** carcinoma showing thymus-like differentiation (CASTLE), thymic carcinoma, next-generation sequencing (NGS), tumor mutational burden (TMB), tumor microenvironment (TME), immunotherapy

## Abstract

Carcinoma showing thymus-like differentiation (CASTLE) is a rare malignancy arising in the thyroid or neck, with an uncertain cellular origin that complicates both diagnosis and treatment. To better understand its molecular underpinnings and identify potential therapeutic avenues, we conducted integrated whole-exome and transcriptome sequencing on six CASTLE and six thymic carcinoma samples. Whole-exome sequencing (WES) was performed on all 12 samples, while RNA sequencing was successful for 1 CASTLE and 6 thymic carcinoma samples. Our analysis included somatic mutation profiling, mutational signature deconvolution, differential gene expression, and characterization of tumor microenvironment for the cases with available data, with comparisons to genomic data from other thyroid cancers. CASTLE tumors demonstrated a higher median tumor mutational burden than thymic carcinoma and lacked the common *BRAF* and *RAS* mutations typically found in thyroid cancers. They harbored alterations in genes such as *TRHDE*, cilia-associated genes (*ANKS6*, *CFAP46*, *DNAH6*), and Wnt signaling components (*TRRAP*, *BCL9L*), as well as mutational signatures suggestive of mismatch repair deficiency and oxidative damage. MSIsensor-pro analysis of the WES data provided support for the potential for mismatch repair deficiency in a subset of CASTLE samples. Exploratory transcriptomic analysis from a single CASTLE case showed downregulation of thyroid follicular markers and an “immune-hot”, lymphocyte-rich microenvironment, closely resembling that of thymic carcinoma. While these findings require validation in larger cohorts, they support a thymic origin for CASTLE and establish its molecular distinction from follicular-derived thyroid cancers. The immunogenic tumor landscape suggests that immune checkpoint inhibitors, particularly those targeting PD-1/PD-L1, may be a promising therapeutic strategy, alongside emerging targets for precision oncology.

## 1. Introduction

Carcinoma showing thymus-like differentiation (CASTLE) is a rare and poorly understood neoplasm arising in the soft tissues of the neck or within the thyroid gland. First reported by Miyauchi et al. in 1985 [1] as “thymus carcinoma in the thyroid”, it was later formally termed “CASTLE” by Chan and Rosai in 1991 [2]. Recognized by the World Health Organization (WHO) in 2004 as a distinct clinicopathological entity of thyroid tumors [3], CASTLE accounts for only about 0.1–0.15% of all thyroid cancers [4]. It is thought to originate from ectopic thymic tissue or branchial pouch remnants within the thyroid gland [5]. Patients typically present with a painless, slowly growing neck mass, though hoarseness or dysphagia may occur due to local invasion into the recurrent laryngeal nerve or esophagus. Clinical and imaging findings often resemble those of other thyroid or thymic malignancies, with no specific distinguishing features [4].

Because of its rarity, optimal treatment strategies for CASTLE remain poorly defined [6]. Surgical resection is commonly performed, but the choice of surgical approach remains largely empirical rather than evidence-based. Although overall prognosis is relatively favorable, aggressive cases with early recurrence and metastasis have been reported [7,8,9,10]. Notably, extrathyroidal extension occurs in 50–60% of cases and lymph node metastasis in about 50%, reflecting its locally invasive potential [8,11]. Upon dissemination, there is also no standard systemic treatment; chemotherapy is the mainstay as it does not accumulate iodine [10].

Diagnosis can be challenging due to histological overlap with lymphoepithelioma, thymic carcinoma, and squamous cell carcinoma [2]. Immunohistochemical staining for CD5 is often used to support the diagnosis, although its specificity is not absolute. While the 2022 WHO classification increasingly incorporates molecular diagnostics for thyroid tumor subtyping, the role of molecular profiling in guiding clinical management (particularly for rare tumors like CASTLE) remains limited.

Next-generation sequencing (NGS) offers a promising approach to identify recurrent genetic alterations in CASTLE and uncover potential therapeutic targets. In this study, we employed NGS to characterize the genomic landscape of CASTLE, aiming to improve our understanding of the molecular underpinnings of this rare malignancy and inform future diagnostic and therapeutic strategies.

## 2. Results

### 2.1. Cohort Characteristics

This retrospective study characterized a cohort of Chinese patients with thymic squamous cell carcinoma (TSCC, *n* = 6) and CASTLE (*n* = 6). Patients were predominantly middle-aged to older adults (with a mean age at diagnosis of 58 years). There was an overall male predominance, more pronounced in the thymic carcinoma group, whereas CASTLE cases showed a more balanced sex distribution. Immunohistochemical data were available for most TSCC and several CASTLE cases. CD5 and CD117 (KIT) expression was frequently observed across TSCC cases and was also present in CASTLE, supporting shared thymic differentiation.

### 2.2. Whole-Exome Somatic Mutation Analysis

**Figure 1:**
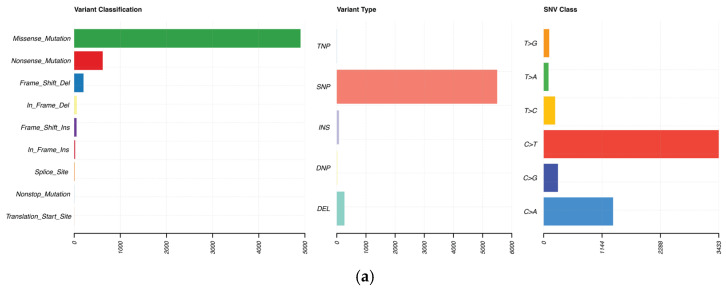

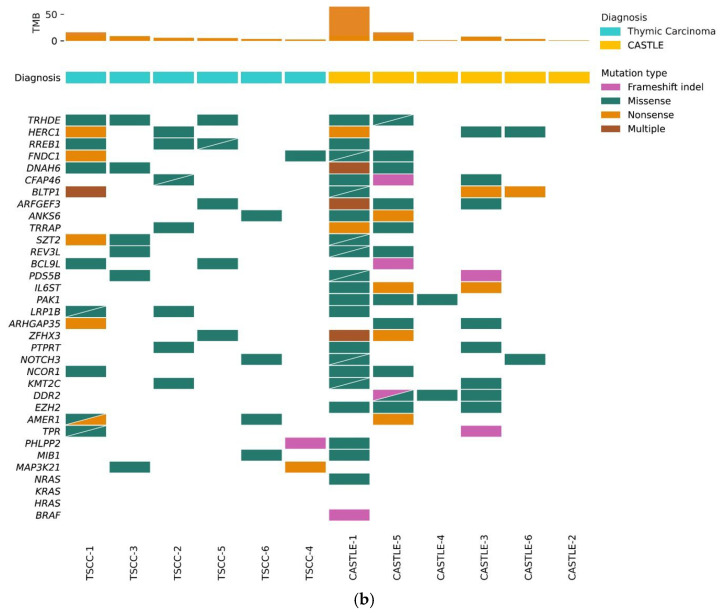
Somatic mutation landscape of the thymic carcinoma (*n* = 6) and CASTLE (*n* = 6) cohorts. (**a**) Overview of somatic variant classification (missense, nonsense, frameshift, etc.) and types across the cohort. (**b**) Co-mutation plot of the 30 most frequently mutated genes (present in at least two samples) alongside canonical MAPK pathway genes (*BRAF*, *NRAS*, *HRAS*, and *KRAS*). Genes are sorted by total mutation frequency and samples are sorted by tumor mutational burden (TMB). Mutation types are color-coded: missense (green), nonsense (orange), frameshift indel (purple), and multiple mutations (brown). Split cells indicate two mutations in the same sample. The histogram above the heatmap shows TMB per sample. TMB = (number of PASS non-synonymous somatic single nucleotide variants and small indels with variant allele frequency ≥ 0.05)/callable exome size (megabase).

WES and RNA sequencing were performed on tumor tissue specimens, in order to characterize the molecular alteration landscape of CASTLE and TSCC. Whole-exome sequencing was performed on all 12 samples (6 CASTLE, 6 TSCC). Using a matched tumor-normal somatic mutation calling pipeline (with a pooled normal sample where matched adjacent tissue was unavailable), we identified 5864 somatic mutations in the exonic regions. Missense mutations accounted for 83.63% of the somatic mutations, while nonsense, frameshift indels, in-frame indels, and splice-site mutations comprised 10.54%, 4.30%, 1.31%, and 0.14%, respectively (Figure 1a). The WES mutation calling results for each sample are summarized in Supplementary Table S1. The samples showed 29 to 2784 somatic mutations in the exome (median 231.5; average 488.7). The median tumor mutational burden (TMB) for our cohort was 5.38 mutations per megabase (mut/Mb). CASTLE samples exhibited a higher median TMB compared to reported values for thymic carcinoma (median TMB = 3.8), aligning more closely with squamous cell carcinoma lineages.

The gene co-mutation plot showed eight genes that were recurrently mutated in at least four patients (33.3%) of our CASTLE and TSCC cohort. These genes may reveal novel pathway insights and biological processes distinct from papillary thyroid carcinoma (PTC). *TRHDE* was mutated in five patients (41.7%). Mutations in *ANKS6*, *CFAP46*, or *DNAH6* were present in seven patients (58.3%), implicating dysregulation of thyroid hormone metabolism, ciliary function, and Wnt/β-catenin signaling. We also observed recurrent mutations in Wnt signaling components *TRRAP* and *BCL9L* (41.7% of cases) (Figure 1b).

The CASTLE and TSCC cohort displayed a somatic mutation profile distinct from common thyroid carcinoma subtypes. While PTCs are frequently driven by mutations in *BRAF*, *NRAS*, *HRAS*, or *KRAS*, these canonical RAS/MAPK pathway alterations were rare in our CASTLE and TSCC cohort (only 1 out of 12 patients, 8.3%). However, we observed that two other MAPK-related genes, *FNDC1* and *RREB1*, which act upstream and downstream of the RAS/MAPK signaling pathway, respectively, were recurrently mutated in six patients (50%) (Figure 1b).

### 2.3. Distinct Profile of CASTLE

**Figure 2:**
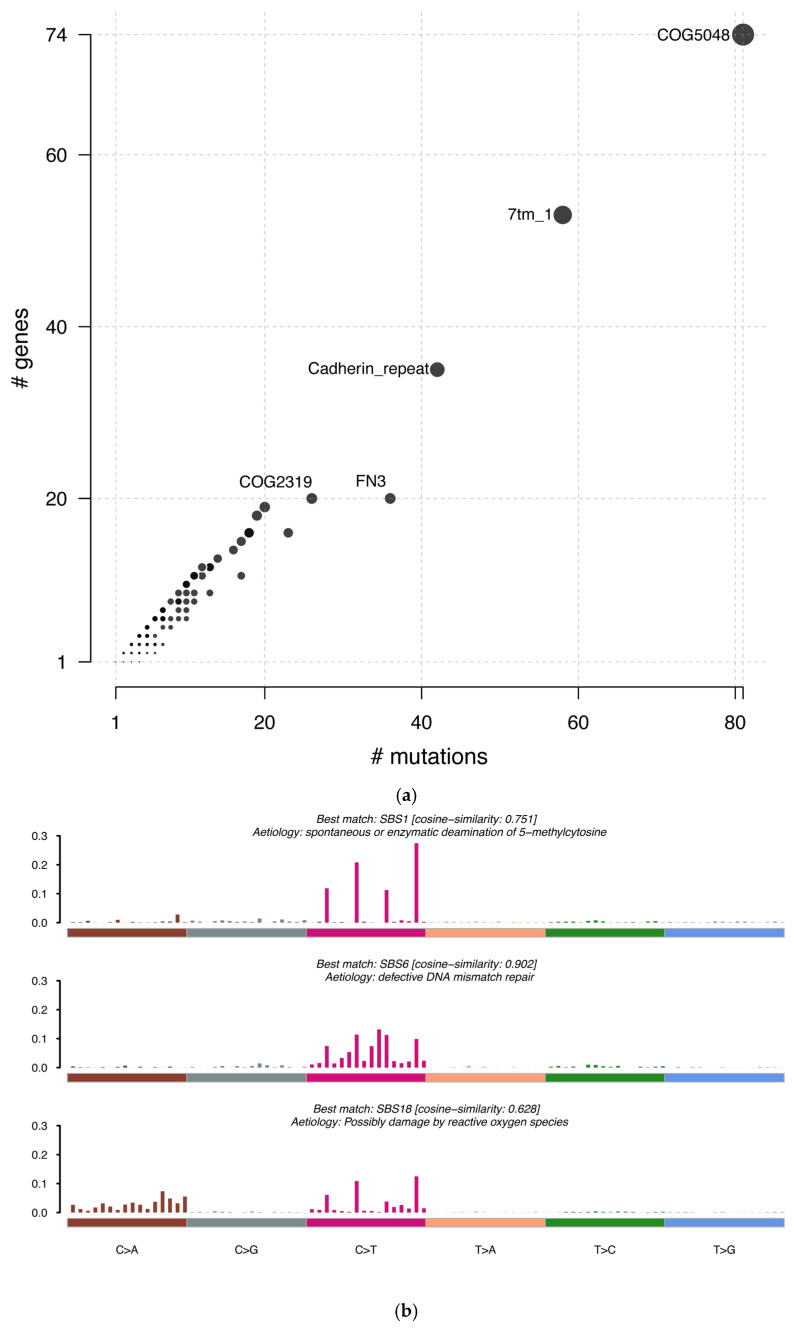
Protein domain mutation enrichment and single-base substitution (SBS) mutational signature analysis in the CASTLE cohort. (**a**) Scatter plot illustrating the enrichment of somatic mutations across specific protein domains. The size of each dot corresponds to the relative abundance of mutations in a protein domain. The y-axis indicates the number of genes harboring a given domain, and the x-axis shows the total mutation count within that domain across the CASTLE cohort. Domains with significant mutation enrichment are labeled. Protein domain mutation enrichment analysis revealed recurrent mutations in cadherin repeats and fibronectin type III (FN3) domains. (**b**) Bar plots illustrate the distribution of SBS across the 96 possible mutation types. The x-axis represents these types, categorized into six substitution classes (C>A, C>G, C>T, T>A, T>C, T>G), each assigned a unique color code (brown, grey, pink, orange, green, and blue, respectively). Within each of the six classes, mutations are further resolved into 16 trinucleotide contexts for the specific bases immediately 5’ and 3’ to the mutated nucleotide. The y-axis indicates the relative contribution of each mutation type to the signature. The three dominant signatures identified (SBS1, SBS6, and SBS18) are shown with their proposed biological etiology and cosine similarity scores relative to the COSMIC/ICGC pan-cancer reference signature catalogue.

Protein domain mutation enrichment analysis revealed recurrent alterations in cadherin repeats and fibronectin type III (FN3) domains in CASTLE, suggesting disrupted cell–cell adhesion and extracellular matrix interactions (Figure 2a).

To explore potential oncogenic processes in CASTLE, we calculated single-base substitution (SBS) signatures using the reference signatures from the ICGC/TCGA pan-cancer analysis [12]. Three mutational signatures were identified. Signature 1 was most similar to SBS1 (associated with endogenous deamination), which is the most frequently observed signature across various cancer types [13]. We also observed an enrichment of SBS6 (which is linked to defective DNA mismatch repair in other cancer types; cosine similarity 0.902) and SBS18 (associated with oxidative damage; cosine similarity 0.628), which were not prominent in the TCGA PTC cohort (Figure 2b). This distinct mutational signature profile in CASTLE may reflect differential oncogenesis mechanisms, further distinguish CASTLE from the typical signature profiles of PTC and broaden our molecular understanding of thyroid cancers.

To evaluate mismatch repair deficiency suggested by mutational signature analysis, MSIsensor-pro was used to check if any samples have microsatellite instability (MSI). MSI scores were interpreted using TCGA-derived thresholds (MSI-H ≥ 10%; MSI-L 3–10%; MSS < 3%). All analyzable CASTLE and TSCC samples fell within the MSI-H range (percentage unstable sites from 12.4% to 16.29%). Consequently, SBS6 observed in the samples may suggest the presence of mismatch repair deficiency.

### 2.4. Exploratory Transcriptome Analysis of a Single CASTLE Reveals Immune-Rich Microenvironment

**Figure 3:**
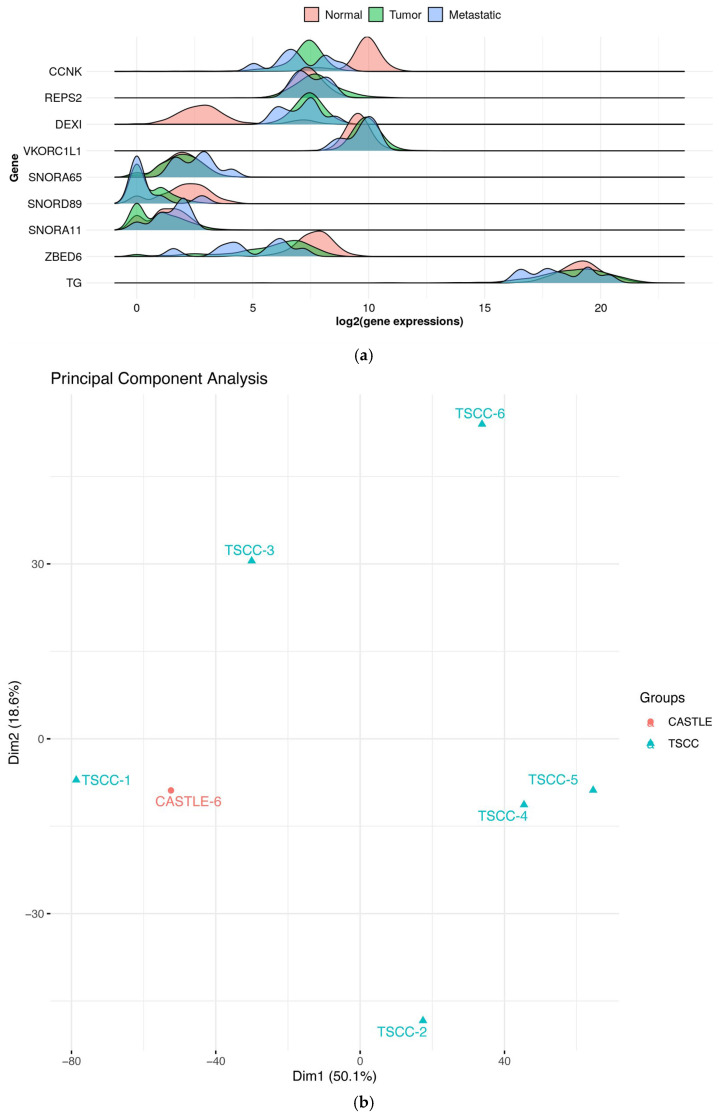
Top differentially expressed genes and transcriptional heterogeneity in thymic carcinoma and a CASTLE case. (**a**) Volcano plot highlighting the most significantly upregulated and downregulated genes in thymic squamous cell carcinoma (TSCC, *n* = 6) and the single CASTLE sample (CASTLE-6) relative to normal thyroid tissue. Expression is shown as log2 fold change (tumor vs. normal). CCNK was the most upregulated gene, while thyroglobulin (*TG*) was the most downregulated in primary tumors. Metastatic samples exhibited a similar but often stronger expression pattern. (**b**) Principal component analysis of RNA-seq data from TSCC (*n* = 6) and the single CASTLE sample (CASTLE-6). Among the samples, CASTLE-6, TSCC-1, TSCC-3, and TSCC-6 were metastatic cases.

Owing to the difficulty of extracting high-quality RNA from the old archival CASTLE samples, successful RNA sequencing was achieved for only one CASTLE patient and all six TSCC patients. The following analyses are therefore exploratory and based on this single CASTLE case. Transcriptome analysis was performed from the tumor tissues in these 6 TSCC patients and the one CASTLE patient. Due to limited RNA availability from archival FFPE tissue, additional CASTLE samples were not available for RNA sequencing. Differential expression analysis was conducted primarily to contextualize the CASTLE sample relative to TSCC tumors and 653 normal thyroid tissues from GTEx. The top 20 differentially expressed genes were identified, with *CCNK* as the most significantly upregulated gene and thyroglobulin (*TG*) as the most significantly downregulated gene (Figure 3a, Supplementary Table S2). Principal component analysis of all expression data revealed two distinct transcriptional clusters among the samples (Figure 3b). Gene terms and pathway enrichment analysis of the top 200 differentially expressed genes exhibited enrichment in the thyroid hormone synthesis pathway according to the KEGG pathway database (adjusted *p* = 0.002).

**Figure 4:**
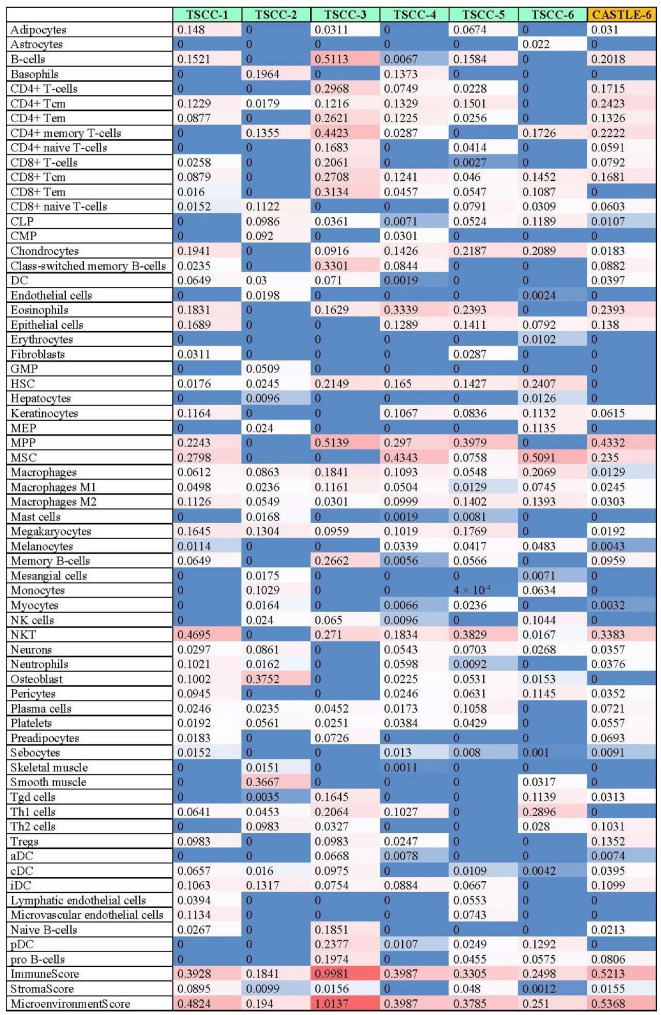
Tumor microenvironment of thymic carcinoma and a CASTLE case. Heatmap of xCell-derived relative immune and stromal cell enrichment scores based on deconvolution of bulk RNA-seq data from thymic carcinoma (*n* = 6) and the single CASTLE sample (CASTLE-6). Rows represent cell types and columns represent individual patients. The top annotation bar indicates diagnosis: thymic carcinoma (teal) and CASTLE (orange). Composite immune, stroma, and microenvironment scores are shown for each sample below the heatmap. In this single case, the CASTLE sample displays a lymphoid-rich, “immune-hot” profile with enrichment scores similar to thymic carcinoma. The normalized enrichment signals of each cell type in each sample calculated by xCell is denoted in each table cell. A continuous color gradient represents relative abundance, where cells highlighted in red color indicate higher abundance, while cells highlighted in red color indicate higher abundance, while cells highlighted in blue indicate low abundance. Lighter shades between red and blue represent intermediate values.

Cell-type enrichment analysis using xCell demonstrated that the CASTLE case exhibited higher relative immune enrichment scores, characterized by a lymphoid lineage infiltration that distinguishes it from the typical landscape of thyroid-derived malignancies. Consistent with its proposed origin from ectopic thymic tissue, the CASTLE sample displayed relative enrichment for an “immune-hot” phenotype comparable to the TSCC samples (Figure 4). Both groups showed enrichment of B-cells and CD4+ T-cells, alongside higher overall immune scores when compared to PTC (median immune score ~0.03) [14]. This lymphocytic infiltration is a defining histological hallmark of CASTLE and is confirmed here at the transcriptomic level. Furthermore, the relatively low scores for cancer-associated fibroblasts (CAFs) and endothelial cells in the CASTLE sample (stroma score = 0.0155) align with the TSCC samples, diverging from the highly desmoplastic and angiogenic stroma frequently observed in aggressive thyroid subtypes like anaplastic thyroid carcinoma [15]. While tumor purity and batch effects may influence the relative enrichment scores returned by xCell, these results may reveal comparative immune signal enrichment and require validation in additional CASTLE cases. Due to tissue-availability limitation, transcriptomic and tumor microenvironment (TME) findings related to CASTLE should be interpreted as a case report rather than representative cohort-level characteristics.

### 2.5. Identification of Potential Therapeutic Targets

Current options for CASTLE treatment are still limited. In search of subjects who may benefit from off-label targeted therapy, we identified two subjects who have a mutation that predicts drug sensitivity according to the OncoKB database [16]. A *TSC2* p.R611Q mutation in a CASTLE patient (CASTLE-6) was associated with predicted sensitivity to ABI-009 and everolimus treatment in perivascular epithelioid cell tumor and glioma, respectively. A *PIK3CA* p.R108H mutation in a TSCC patient (TSCC-1) predicted sensitivity to alpelisib + fulvestrant and capivasertib + fulvestrant in other cancer contexts, suggesting avenues for exploration (Table 1). The SBS6 mutational signature identified in CASTLE, along with supporting evidence from MSIsensor-pro analysis, suggests a subset of these tumors may harbor defects in DNA repair, which could be linked with sensitivity towards platinum-based chemotherapy. The immune-hot microenvironment observed in the single CASTLE case suggests a potential role for immunotherapy, an approach that has demonstrated clinical efficacy in histologically similar malignancies, including squamous cell carcinoma and thymic carcinoma. These potentially actionable mutations should be considered exploratory observations requiring orthogonal validation prior to therapeutic interpretation, as subclonal events or FFPE-associated artifacts may produce false-positive calls.

## 3. Discussion

This integrated molecular analysis characterizes the distinct genomic and microenvironmental landscape of CASTLE and TSCC. The median TMB in our cohort was 5.38 mut/Mb. In comparison, a recent real-world study reported a median TMB of 3.8 mut/Mb for thymic carcinoma, with the squamous cell carcinoma subtype showing the highest burden, though only 9% of those cases reached a TMB ≥ 20 mut/Mb [17]. While CASTLE shares histological features with both squamous and thymic lineages, its elevated TMB aligns more closely with squamous cell carcinoma and distinguishes it genomically from typical thymic carcinoma, further supporting CASTLE’s unique molecular identity. In our cohort, two out of six CASTLE samples exhibit TMB > 10 mut/Mb, which enables the use of pembrolizumab under current tumor-agnostic FDA indication [18].

The somatic mutation profile of CASTLE and TSCC differs substantially from that of conventional thyroid carcinoma. Recurrent mutations were identified in genes linked to distinct biological pathways. *TRHDE*, mutated in over 40% of our cohort, encodes an enzyme that degrades thyrotropin-releasing hormone. Alterations in this gene may dysregulate thyroid-stimulating hormone levels, indirectly affecting thyrocyte proliferation and differentiation (a hypothesis supported by our RNA-seq data showing significant differential expression in thyroid hormone synthesis pathways). Additionally, cilia-related genes (*ANKS6*, *CFAP46*, *DNAH6*) were mutated in 58.3% of patients. Dysfunctional cilia could disrupt key signaling pathways such as Hedgehog or Wnt, both implicated in thyroid differentiation [19]. We also observed recurrent mutations in *TRRAP* and *BCL9L* (41.7% of cases), which are involved in Wnt/β-catenin and PI3K-AKT signaling [20,21]. Together, these findings point to unique oncogenic mechanisms in CASTLE involving thyroid hormone metabolism, cellular signaling, and ciliary function. A potential future direction to reinforce these biological conclusions is the validation of findings through in vitro or in vivo studies.

Notably, our CASTLE samples lacked the canonical driver mutations common in other thyroid cancers. Whereas PTC frequently harbor RAS/MAPK pathway genes (such as *BRAF*, *NRAS*, *HRAS*, and *KRAS*) [17,22,23,24], these alterations were present in only 8.3% of our patients. Our findings contrast with the TCGA PTC cohort, where kinase domain alterations (e.g., *BRAF*, *RAS*) were most prevalent. This absence underscores a divergent pathogenic pathway. Further supporting this distinction, protein domain analysis revealed an enrichment of mutations in cadherin repeats and FN3 domains, suggesting that loss of cellular adhesion may be a critical oncogenic event in CASTLE, contrasting with the kinase-driven pathogenesis of PTC. Despite our cohort’s small size, these molecular differences highlight the heterogeneity among thyroid carcinoma subtypes and indicate that CASTLE may rely on alternative oncogenic mechanisms.

Mutational signature analysis provided additional insights into potential etiologic factors. We identified a strong contribution from the SBS6 signature (which is linked to mismatch repair deficiency in other cancer types). This, along with supporting evidence from MSIsensor-pro analysis, suggests a subset of CASTLE tumors may harbor defects in DNA repair. This has potential implications for treatment with platinum-based chemotherapy or immunotherapy, but requires functional validation. The presence of the SBS18 signature (linked to oxidative damage) further distinguishes CASTLE from PTC and points to a role for oxidative stress in its tumorigenesis. Combined with a TMB profile that resembles squamous cell carcinoma more than thymic carcinoma, these signatures underscore the molecular hybridity of CASTLE and its distinct pathogenic journey. These findings are consistent with the clinically observed sensitivity towards platinum-based chemotherapy.

*TG* was the most significantly downregulated genes compared with GTEx normal thyroid tissue in the transcriptome analysis. Given that *TG* is a canonical marker of differentiated thyroid follicular cells and its expression level is the highest in normal thyroids [25], its reduction in thyroid carcinoma samples may reflect dedifferentiation of cancer cells from their thyroid follicular lineage [26,27].

In the exploratory transcriptome and TME analysis of a single CASTLE case, the CASTLE sample displayed an “immune-hot”, lymphocyte-rich TME closely resembling that of thymic carcinoma, characterized by high immune scores and minimal stromal components. This pattern is not typical of thyroid-derived malignancies and provides preliminary molecular support for the histological classification of CASTLE as a thymic-like entity arising from ectopic tissue. Given the preliminary, case-level nature of this finding, it requires validation through orthogonal methods such as immunohistochemistry or quantitative PCR in future studies with larger cohorts. Clinically, this suggests that immunotherapeutic strategies effective in thymic carcinoma and squamous cell carcinoma (particularly immune checkpoint inhibitors targeting the PD-1/PD-L1 axis) may also be relevant for CASTLE.

We also identified potentially actionable mutations (such as those in *TSC2* and *PIK3CA*) as observations, highlighting the value of comprehensive molecular profiling for CASTLE and TSCC. While functional or pharmacological validation is required to support therapeutic recommendations, and orthogonal confirmation of these low-variant allele frequency (VAF) variants is necessary, these alterations offer a hypothesis-generating rationale for exploring targeted therapies in selected patients.

Indeed, the rarity of CASTLE has hindered a systematic understanding of its molecular drivers and therapeutic vulnerabilities. Nevertheless, case reports are beginning to suggest that targeted therapies (especially immune checkpoint blockade) could offer benefit in advanced disease. In a case report of parotid CASTLE, a patient with metastatic disease achieved a partial response to anti-PD-1 therapy (pembrolizumab) [28]. Similarly, another case report of a patient with metastatic salivary gland CASTLE also partially responded to nivolumab [29]. These early clinical signals point to a biologically relevant role for the PD-1/PD-L1 axis in at least a subset of CASTLEs and emphasize the pressing need for comprehensive genomic profiling in this disease. Through systematic molecular characterization, we may identify predictive biomarkers, uncover resistance mechanisms, and discover new actionable targets, ultimately paving the way for more personalized management of this rare malignancy.

Our study has several limitations, including its retrospective design, small sample size (inherent to the rarity of CASTLE), and reliance on archival FFPE specimens, absence of matched normal tissue for some samples, as well as issues related to tumor purity and stromal contamination. The small cohort size is a significant limitation. Therefore, the meaningful findings should be considered preliminary or hypothesis-generating to minimize the risk of overinterpretation. Furthermore, successful RNA sequencing was achieved for only one of the six CASTLE samples, meaning the transcriptomic and tumor microenvironment analyses are based on a single case. Consequently, these findings must be regarded as exploratory, case-level observations that are hypothesis-generating and require validation in future, larger-scale studies using orthogonal methods like immunohistochemistry or quantitative PCR. While we employed state-of-the-art methods to mitigate fixation artifacts, the possibility of some artifactually elevated TMB in older samples cannot be entirely excluded. The CASTLE samples were archived longer than the rest of the cohort. It is plausible that the archival age of FFPE blocks contributed to some artifactual mutation calls; however, studies indicated that storage duration alone is not expected to generate a two-fold or higher increase in somatic mutation counts. Prior work demonstrated that archival FFPE specimens yield reproducible NGS data even after many years of storage, with quality metrics suitable for genomic research and without systematic artifactual mutation inflation attributable solely to storage time [30]. Formalin fixation induces characteristic artifactual substitutions, predominantly C>T transitions from cytosine deamination, which are mitigated by enzymatic repair in our DNA extraction and sequencing library preparation workflows, as well as computational mutation filtering strategies [31]. While some artifactually elevated calls cannot be entirely ruled out, the overall magnitude and consistency of the mutation burden observed are unlikely to be explained by storage-related artifacts alone. To further investigate this, we stratified the fraction of C>T transitions by sample age and by matched-normal vs. pooled-normal processing (Supplementary Figure S1). The biological coherence of our findings across genomic and transcriptomic platforms supports their validity. Matched normal tissue was unavailable for some CASTLE samples, which may affect the accuracy of mutation calling. In cases where patient-matched tumor-adjacent samples were not available, we employed pooled tumor-adjacent samples that are highly correlated with the tumor site to serve as controls, while acknowledging that adjacent normal tissues may exhibit transcriptomic profiles influenced by the TME. The immune-enriched transcriptional features were based on a single CASTLE case. This observation does not establish an intrinsic CASTLE immune phenotype but aligns with prior histological reports and supports further investigation. Additionally, tumor purity and stromal contamination may impact both mutation and expression profiles. To address these challenges, we applied a combination of computational, experimental, and statistical approaches to effectively mitigate their effects, thereby improving the accuracy of our molecular analyses.

## 4. Materials and Methods

### 4.1. Sample Collection

Formalin-fixed paraffin-embedded (FFPE) tissue sections from patients diagnosed with CASTLE or thymic carcinoma were obtained from the archives of Queen Elizabeth Hospital (Hong Kong SAR, China) between 1 January 1990 and 31 December 2022. The diagnosis of CASTLE was confirmed by CD5 immunohistochemistry. A total of 8 CASTLE and 6 TSCC (as control) tissue samples were selected for NGS. Two CASTLE samples were subsequently excluded due to insufficient DNA/RNA quality.

### 4.2. DNA and RNA Extraction

To explore the genomic and transcriptomic alterations, both DNA and RNA were extracted from homogenized tissue samples using the Qiagen AllPrep DNA/RNA FFPE Kit (Qiagen, Venlo, The Netherlands) following the manufacturer’s recommendations.

### 4.3. Whole-Exome Sequencing (WES) and Bioinformatic Analysis

We sequenced the genomic DNA and RNA of CASTLE and thymic tumor samples using WES technology. Extracted DNA was enzymatically fragmented to approximately 250 bp using the KAPA frag kit (Roche, Rotkreuz, Switzerland). Whole-exome enrichment was performed using the KAPA Hyperexome v1 Probe (Roche, Rotkreuz, Switzerland). Sequencing library preparation and target enrichment were carried out according to the manufacturer’s instructions. Library quality was assessed using the Tapestation 4150 (Agilent, Santa Clara, CA, USA) and Qubit 4 fluorometer (Thermo Fisher, Waltham, MA, USA), with only libraries showing a peak fragment size between 200 and 300 bp and concentration ≥ 20 ng/µL proceeding to sequencing. High-throughput sequencing was performed using the Illumina NovaSeq Plus X PE150 platform (Illumina, San Diego, CA, USA). Tumor samples were sequenced at an average raw sequencing depth of 405.4×, while matched normal samples were sequenced at an average depth of 224.2× (Supplementary Table S3). High sequencing quality was achieved, where the per-base quality scores were ≥ Q30 for >90% of bases according to FastQC.

Sequencing reads were aligned using dragmap (Illumina) to ensure accurate mapping to the graph-based hg38 human reference genome. Additionally, duplicate reads were marked using GATK 4.4 [32] to mitigate PCR biases introduced during library preparation and sequencing. The average sequence alignment rate was 99.7% and the sequence duplication rate was 26.7% according to GATK Picard. VerifyBamID was used to verify that no inter-sample contamination was found.

Mutect2 [32] was employed for tumor-normal single-nucleotide variant (SNV) and insertion/deletion (indel) calling. We used the matched tumor-normal somatic mutation calling pipeline to get SNVs, indels, CNVs, gene fusions, and copy number alterations. To navigate the challenge of obtaining tissue-adjacent specimens, a pooled normal sample was used instead if a matching tumor-adjacent sample was not available. The list of samples that have matched normal is indicated in Supplementary Table S4. A panel of normal samples based on the gnomAD and 1000 Genomes Project was obtained from the Mutect2 resource bundle, so that germline mutations with population allele frequency > 1% were removed. Recommended filtering parameters from Mutect2 best practices were followed, and only mutations that pass FilterMutectCalls and have a VAF > 0.05 are reported. Somatic variant filtering was performed using GATK FilterMutectCalls with FFPE-optimized parameters. Variants were excluded based on the following filter categories: weak evidence (allele limit of detection below adaptive threshold), strand bias, cross-sample contamination (GetPileupSummaries/CalculateContamination), FFPE/OxoG read-orientation artifacts learned via LearnReadOrientationModel, microsatellite slippage, haplotype in-consistency, and germline evidence using the 1000 Genomes panel of normals. Per-sample PASS rates, callable exome sizes, and filter-specific false-positive estimates are provided in Supplementary Table S4. Tumor mutational burden (TMB) was computed as follows: TMB = (Number of PASS non-synonymous somatic SNVs and small indels with VAF ≥ 0.05)/callable exome size (Mb).

Callable exome size was derived per sample from Mutect2 statistics files (mean ≈ 33.5 Mb; target capture: HyperExome v1, 35.794 Mb). Variants failing FilterMutectCalls were excluded. MSIsensor-pro (v1.3+) was applied in tumor-only mode using a GRCh38 microsatellite baseline. MSI scores were interpreted using established thresholds (MSI-H ≥ 10%; MSS < 10%). The mutations were annotated into mutation annotation format (MAF) files using vcf2maf. The MAF files were further filtered to remove synonymous and noncoding mutations, followed by summarization using default maftools parameters [33]. Single-base substitution signatures were extracted using SigProfilerExtractor 1.1.23 [12]. Furthermore, Manta [34] was utilized to detect structural variants such as chromosomal rearrangements and large-scale indels. Lastly, CNVkit [35] enabled assessment of copy number variations (CNVs), providing insights into gene dosage alterations. Quality checking of sequencing data, alignment, and variant calling were performed using FastQC 0.12.1, GATK 4.5.0.0, Picard 3.1.1, SAMtools 1.19.2, and BCFtools 1.19 [36].

### 4.4. RNA Sequencing and Transcriptomic Analysis

Total RNA isolated from tumor samples was assessed using Tapestation 4150 (Agilent) and Qubit 4 fluorometer (Thermo Fisher) to ensure that the quantity and integrity met the recommended input requirements for downstream lab processes. Subsequently, the NEBNext^®^ rRNA Depletion Kit v2 (New England Biolabs, Hitchin, UK) was used to selectively removed ribosomal RNAs (rRNAs), allowing us to focus on genic transcripts. The resulting RNA was reverse-transcribed into cDNA using the PrimeScript™ Double Strand cDNA synthesis kit (Takara, Shiga, Japan). For sequencing library construction, the KAPA HyperPlus Kit (Roche) was used, followed by high-throughput sequencing on the NovaSeq Plus X PE150 platform (Illumina). High sequencing quality was achieved, where the per-base quality scores were ≥ Q30 for >90% of bases according to FastQC.

Low-quality bases were trimmed from the RNA-seq reads using Cutadapt [37]. The trimmed reads were aligned to the human reference genome (version hg38) using a graph-based transcriptome aligner called HISAT2 [38]. The sequence alignment rate was 100% and the sequence duplication rate was 79.2% according to GATK Picard. Annotations from GENCODE v45 [39] were leveraged to provide gene-, transcript-, and exon-level information. Reads mapped to protein-coding regions and lncRNA regions were >80%, while the average amount of rRNA is <5% of reads, indicating adequate RNA sequencing quality. Differential gene expression analysis using DESeq2 [40] allowed for the detection of differentially expressed genes in the cancer tissue compared to normal thyroid tissue expression profiles from the GTEx database [25]. To unravel the biological context, pathway and gene set enrichment analysis was performed using the published gprofiler2 0.2.3 package [41]. The significance of these pathways was assessed based on statistical thresholds (*p* < 0.05). The xCell 1.1.0 R 4.2.2 package was used to deconvolute the cell type abundance in the normalized bulk RNA-seq transcriptome data [42].

Fusion genes have emerged as critical players in cancer pathogenesis. To explore this aspect, three fusion gene detection tools (Arriba 2.4.0 [43], FusionCatcher 1.33, and STAR-Fusion 1.13.0 [44]) were employed. These tools scrutinize RNA-seq data for chimeric transcripts resulting from gene fusions.

### 4.5. Statistical Analysis

Rstudio 2023.12.1, Maftools 2.14.0, Python 3.12.1, Numpy 1.26.3, and Scipy 1.11.4 were used for statistical analysis. Results are described in descriptive statistics. Unless otherwise specified, a two-tailed *p*-value less than 0.05 was considered statistically significant.

## 5. Conclusions

In summary, CASTLE is molecularly distinct from thyroid follicular-derived carcinoma and shares key microenvironmental features with thymic carcinoma. Our data support its classification as a thymic epithelial tumor and provide a molecular rationale for considering immunotherapy, especially in cases with high TMB. The transcriptomic findings are based on a single case and are therefore exploratory and hypothesis-generating, requiring validation in future studies. The identification of recurrent alterations in hormone-regulating, ciliary, and signaling pathways opens new avenues for mechanistic research and identification of therapeutic targets. These findings enhance our understanding of CASTLE biology and expand the potential treatment landscape for this rare malignancy.

## Figures and Tables

**Table 1:** Potential observations of druggable mutations.

Sample	Gene	HGVS	Consequence	VAF	Potential Sensitive Drug
CASTLE-6	*TSC2*	c.1832G>A p.R611Q	missense_ variant	0.076	ABI-009, Everolimus
TSCC-1	*PIK3CA*	c.323G>A p.R108H	missense_ variant	0.06	Alpelisib + Fulvestrant, Capivasertib + Fulvestrant

Note: Variants are listed with low variant allele frequencies (VAFs) and should be considered observations requiring orthogonal confirmation (e.g., by amplicon sequencing). Therapeutic implications are extrapolated from other cancer contexts and are hypothesis-generating.

## Data Availability

The data that support the findings of this study are available from the corresponding author on request.

## Supplementary Materials

The following supporting information can be downloaded at https://www.mdpi.com/article/10.3390/ijms27083501/s1.

## Abbreviations

The following abbreviations are used in this manuscript:

CAF	Cancer-associated fibroblast
CASTLE	Carcinoma showing thymus-like differentiation
CNV	Copy number variation
FFPE	Formalin-fixed paraffin-embedded
FN3	Fibronectin type III
Indel	Insertion/deletion
MAF	Mutation annotation format
MSI	Microsatellite instability
mut/Mb	Mutations per megabase
NGS	Next-generation sequencing
PTC	Papillary thyroid carcinoma
QC	Quality control
rRNA	Ribosomal RNA
SNV	Single nucleotide variant
TG	Thyroglobulin
TMB	Tumor mutational burden
TME	Tumor microenvironment
TSCC	Thymic squamous cell carcinoma
VAF	Variant allele frequency
WES	Whole-exome sequencing
WHO	World Health Organization

## References

[1] Miyauchi A., Kuma K., Matsuzuka F., Matsubayashi S., Kobayashi A., Tamai H., Katayama S. (1985). Intrathyroidal epithelial thymoma: An entity distinct from squamous cell carcinoma of the thyroid. World J. Surg..

[2] Chan J.K., Rosai J. (1991). Tumors of the neck showing thymic or related branchial pouch differentiation: A unifying concept. Hum. Pathol..

[3] Cheuk W., Chan J.K.C., Dorfmann D.M., Giordano T., DeLellis R.A., Lloyd R.V., Heitz P.U., Eng C. (2004). Spindle cell tumor with thymus-like differentiation. Pathology and Genetics of Tumours of Endocrine Organs.

[4] Dang N.V., Son L.X., Hong N.T.T., Nhung N.T.T., Tung N.T., Quang L.V. (2021). Recurrence of carcinoma showing thymus-like differentiation (CASTLE) involving the thyroid gland. Thyroid Res..

[5] Lominska C., Estes C.F., Neupane P.C., Shnayder Y., TenNapel M.J., O’Neil M.F. (2017). CASTLE thyroid tumor: A case report and literature review. Front. Oncol..

[6] Luo C.-M., Hsueh C., Chen T.-M. (2005). Extrathyroid carcinoma showing thymus-like differentiation (CASTLE) tumor-A new case report and review of literature. Head Neck.

[7] Chow S.M., Chan J.K.C., Tse L.L.Y., Tang D.L.C., Ho C.M., Law S.C.K. (2007). Carcinoma showing thymus-like element (CASTLE) of thyroid: Combined modality treatment in 3 patients with locally advanced disease. Eur. J. Surg. Oncol..

[8] Ito Y., Miyauchi A., Nakamura Y., Miya A., Kobayashi K., Kakudo K. (2007). Clinicopathologic significance of intrathyroidal epithelial thymoma/carcinoma showing thymus-like differentiation: A collaborative study with Member Institutes of The Japanese Society of Thyroid Surgery. Am. J. Clin. Pathol..

[9] Tsutsui H., Hoshi M., Kubota M., Suzuki A., Nakamura N., Usuda J., Shibuya H., Miyajima K., Ohira T., Ito K. (2013). Management of thyroid carcinoma showing thymus-like differentiation (CASTLE) invading the trachea. Surg. Today.

[10] Hanamura T., Ito K., Uehara T., Fukushima T., Sasaki S., Koizumi T. (2015). Chemosensitivity in carcinoma showing thymus-like differentiation: A case report and review of the literature. Thyroid.

[11] Sun T., Wang Z., Wang J., Wu Y., Li D., Ying H. (2011). Outcome of radical resection and postoperative radiotherapy for thyroid carcinoma showing thymus like differentiation. World J. Surg..

[12] Alexandrov L.B., Kim J., Haradhvala N.J., Huang M.N., Ng A.W.T., Wu Y., Boot A., Covington K.R., Gordenin D.A., Bergstrom E.N. (2020). The repertoire of mutational signatures in human cancer. Nature.

[13] Alexandrov L.B., Nik-Zainal S., Wedge D.C., Aparicio S.A.J.R., Behjati S., Biankin A.V., Bignell G.R., Bolli N., Borg A., Børresen-Dale A.-L. (2013). Signatures of mutational processes in human cancer. Nature.

[14] Na K.J., Choi H. (2018). Immune landscape of papillary thyroid cancer and immunotherapeutic implications. Endocr. Relat. Cancer.

[15] Han P.-Z., Ye W.-D., Yu P.-C., Tan L.-C., Shi X., Chen X.-F., He C., Hu J.-Q., Wei W.-J., Lu Z.W. (2024). A distinct tumor microenvironment makes anaplastic thyroid cancer more lethal but immunotherapy sensitive than papillary thyroid cancer. JCI Insight.

[16] Suehnholz S.P., Nissan M.H., Zhang H., Kundra R., Nandakumar S., Lu C., Carrero S., Dhaneshwar A., Fernandez N., Xu B.W. (2024). Quantifying the expanding landscape of clinical actionability for patients with cancer. Cancer Discov..

[17] Girard N., Basse C., Schrock A., Ramkissoon S., Killian K., Ross J.S. (2022). Comprehensive genomic profiling of 274 thymic epithelial tumors unveils oncogenic pathways and predictive biomarkers. Oncologist.

[18] Marcus L., Fashoyin-Ajie L.A., Donoghue M., Yuan M., Rodriguez L., Gallagher P.S., Philip R., Ghosh S., Theoret M.R., Beaver J.A. (2021). FDA approval summary: Pembrolizumab for the treatment of tumor mutational burden-high solid tumors. Clin. Cancer Res..

[19] Jing J., Wu Z., Wang J., Luo G., Lin H., Fan Y., Zhou C. (2023). Hedgehog signaling in tissue homeostasis.; cancers, and targeted therapies. Signal Transduct. Target. Ther..

[20] Adachi S., Jigami T., Yasui T., Nakano T., Ohwada S., Omori Y., Sugano S., Ohkawara B., Shibuya H., Nakamura T. (2004). Role of a BCL9-related beta-catenin-binding protein, B9L, in tumorigenesis induced by aberrant activation of Wnt signaling. Cancer Res..

[21] Detilleux D., Raynaud P., Pradet-Balade B., Helmlinger D. (2022). The TRRAP transcription cofactor represses interferon-stimulated genes in colorectal cancer cells. Elife.

[22] Kimura E.T., Nikiforova M.N., Zhu Z., Knauf J.A., Nikiforov Y.E., Fagin J.A. (2003). High prevalence of *BRAF* mutations in thyroid cancer: Genetic evidence for constitutive activation of the RET/PTC-RAS-BRAF signaling pathway in papillary thyroid carcinoma. Cancer Res..

[23] Cohen Y., Xing M., Mambo E., Guo Z., Wu G., Trink B., Beller U., Westra W.H., Ladenson P.W., Sidransky D. (2003). *BRAF* mutation in papillary thyroid carcinoma. J. Natl. Cancer Inst..

[24] Cancer Genome Atlas Research Network (2014). Integrated genomic characterization of papillary thyroid carcinoma. Cell.

[25] GTEx Consortium (2013). The Genotype-Tissue Expression (GTEx) project. Nat. Genet..

[26] Suzuki K., Mori A., Lavaroni S., Ulianich L., Miyagi E., Saito J., Nakazato M., Pietrarelli M., Shafran N., Grassadonia A. (1999). Thyroglobulin regulates follicular function and heterogeneity by suppressing thyroid-specific gene expression. Biochimie.

[27] Ibrahimpasic T., Ghossein R., Carlson D.L., Nixon I.J., Palmer F.L., Patel S.G., Tuttle R.M., Shaha A., Shah J.P., Ganly I. (2015). Undetectable thyroglobulin levels in poorly differentiated thyroid carcinoma patients free of macroscopic disease after initial treatment: Are they useful?. Ann. Surg. Oncol..

[28] Lorenz L., von Rappard J., Arnold W., Mutter N., Schirp U., Scherr A., Jehle A.W. (2019). Pembrolizumab in a patient with a metastatic CASTLE tumor of the parotid. Front. Oncol..

[29] Yamamoto H., Kusafuka K., Nozaki Y., Iwasaki T., Nogami M., Hongo T., Yasumatsu R., Oda Y. (2021). Carcinoma showing thymus-like differentiation (CASTLE) of the salivary gland: Report of 2 cases of a hitherto under-recognized extrathyroid counterpart. Pathol. Res. Pract..

[30] Carrick D.M., Mehaffey M.G., Sachs M.C., Altekruse S., Camalier C., Chuaqui R., Cozen W., Das B., Hernandez B.Y., Lih C.-J. (2015). Robustness of next generation sequencing on older formalin-fixed paraffin-embedded tissue. PLoS ONE.

[31] Guo Q., Lakatos E., Bakir I.A., Curtius K., Graham T.A., Mustonen V. (2022). The mutational signatures of formalin fixation on the human genome. Nat. Commun..

[32] Auwera G.A.V., Carneiro M.O., Hartl C., Poplin R., Angel G.D., Levy-Moonshine A., Jordan T., Shakir K., Roazen D., Thibault J. (2013). From FastQ data to high confidence variant calls: The Genome Analysis Toolkit best practices pipeline. Curr. Protoc. Bioinform..

[33] Mayakonda A., Lin D.-C., Assenov Y., Plass C., Koeffler H.P. (2018). Maftools: Efficient and comprehensive analysis of somatic variants in cancer. Genome Res..

[34] Chen X., Schulz-Trieglaff O., Shaw R., Barnes B., Schlesinger F., Källberg M., Cox A.J., Kruglyak S., Saunders C.T. (2016). Manta: Rapid detection of structural variants and indels for germline and cancer sequencing applications. Bioinformatics.

[35] Talevich E., Shain A.H., Botton T., Bastian B.C. (2016). CNVkit: Genome-wide copy number detection and visualization from targeted DNA sequencing. PLoS Comput. Biol..

[36] Danecek P., Bonfield J.K., Liddle J., Marshall J., Ohan V., Pollard M.O., Whitwham A., Keane T., McCarthy S.A., Davies R.M. (2021). Twelve years of SAMtools and BCFtools. Gigascience.

[37] Kechin A., Boyarskikh U., Kel A., Filipenko M. (2017). cutPrimers: A new tool for accurate cutting of primers from reads of targeted next generation sequencing. J. Comput. Biol..

[38] Kim D., Paggi J.M., Park C., Bennett C., Salzberg S.L. (2019). Graph-based genome alignment and genotyping with *HISAT2* and *HISAT*-genotype. Nat. Biotechnol..

[39] Harrow J., Frankish A., Gonzalez J.M., Tapanari E., Diekhans M., Kokocinski F., Aken B.L., Barrell D., Zadissa A., Searle S. (2012). GENCODE: The reference human genome annotation for The ENCODE Project. Genome Res..

[40] Love M.I., Huber W., Anders S. (2014). Moderated estimation of fold change and dispersion for RNA-seq data with DESeq2. Genome Biol..

[41] Kolberg L., Raudvere U., Kuzmin I., Adler P., Vilo J., Peterson H. (2023). g:Profiler-interoperable web service for functional enrichment analysis and gene identifier mapping (2023 update). Nucleic Acids Res..

[42] Aran D. (2020). Cell-type enrichment analysis of bulk transcriptomes using xCell. Methods Mol. Biol..

[43] Uhrig S., Ellermann J., Walther T., Burkhardt P., Fröhlich M., Hutter B., Toprak U.H., Neumann O., Stenzinger A., Scholl C. (2021). Accurate and efficient detection of gene fusions from RNA sequencing data. Genome Res..

[44] Haas B.J., Dobin A., Li B., Stransky N., Pochet N., Regev A. (2019). Accuracy assessment of fusion transcript detection via read-mapping and de novo fusion transcript assembly-based methods. Genome Biol..

