# Supplementary material for: The Molecular Landscape of CASTLE: A Rare Thymus-like Head and Neck Cancer
Source: Int J Mol Sci. 2026 Apr 14;27(8):3501. doi: 10.3390/ijms27083501 (PMC13116657; doi:10.3390/ijms27083501)
Supplement: Supplementary file 1 [file ijms-27-03501-s001.zip › Supplementary Tables.pdf]

Supplementary Tables

Supplementary Table S1. Somatic mutation calling statistics.

Sample	Frameshift Indels	In Frame Indels	Missense	Nonsense	Nonstop	Splice Site	Translation Start Site	Total	TMB
CASTLE-1	61	9	2407	306	1	0	0	2784	64.74
*CASTLE-5	93	45	464	83	0	4	1	690	16.05
*CASTLE-3	18	2	298	25	0	1	0	344	8.00
*CASTLE-6	0	0	145	12	0	0	0	157	3.65
CASTLE-4	2	0	46	6	0	0	0	54	1.26
CASTLE-2	1	0	23	5	0	0	0	29	0.67
TSCC-1	18	4	577	88	1	1	0	689	16.02
TSCC3	17	4	327	37	2	2	0	389	9.05
TSCC2	11	4	207	21	0	0	0	243	5.65
TSCC5	8	3	189	20	0	0	0	220	5.12
TSCC6	13	3	133	9	0	0	0	158	3.67
TSCC4	10	3	88	6	0	0	0	107	2.49

The left Sample column indicates diagnosis: CASTLE (orange) and thymic carcinoma (teal). *Matched tumor-adjacent sample is not available for patients and thus pooled tumor-adjacent sample was used instead. TMB: tumor mutational burden.

Supplementary Table S2. Top 20 most significant differentially expressed genes. The comparison of tumor samples (1 CASTLE and 6 TSCC) against normal control samples (normal thyroid from the GTEx project).

Gene	Gene description	$\log_2$ fold change (vs normal GTEx samples)	*p* value	padj
CCNK	Cyclin K	4.35345439	0	0
REPS2	RALBP1 associated Eps domain containing 2	7.04022537	0	0
DEXI	Dexi homolog	10.1116472	0	0
VKORC1L1	Vitamin K epoxide reductase complex subunit 1 like 1	5.03936885	0	0
SNORA21	Small nucleolar RNA, H/ACA box 21	9.30396924	0	0
SNORA65	Small nucleolar RNA, H/ACA box 65	9.91033966	0	0
SNORA62	Small nucleolar RNA, H/ACA box 62	10.1310649	0	0
SNORD89	Small nucleolar RNA, C/D box 89	9.76995083	0	0
SNORA11	Small nucleolar RNA, H/ACA box 11	9.70038562	0	0
N/A	Novel transcript	9.5292257	0	0
ZBED6	Zinc finger BED-type containing 6	9.62450201	0	0
ZFHX3-AS1	ZFHX3 antisense RNA 1	11.4816458	0	0
N/A	Novel transcript	6.28496143	0	0
TMEM185A	Transmembrane protein 185A	3.71898518	0	0
SNORA61	Small nucleolar RNA, H/ACA box 61	8.74621916	0	0
RN7SL2	RNA component of signal recognition particle 7SL2	11.133867	7.99e-304	2.31e-300
N/A	Novel transcript	7.06872516	9.62e-297	2.62e-293
TG	Thyroglobulin	-11.404888	5.67e-294	1.46e-290
SNORD33	Small nucleolar RNA, C/D box 33	12.6599287	3.30e-287	8.05e-284

Supplementary Table S3. Detailed sequencing and statistics. The left Sample column indicates diagnosis: CASTLE (orange) and thymic carcinoma (teal).

Sample	Lab Code	Tissue Type	Sample Type	Reads	Yield (Gbases)	% Mapped
CASTLE-1	CGLAB20235410	Tumor	DNA	51,906,926	15.57	99.6
CASTLE-1	CGLAB20246468	Tumor-adjacent	DNA	17,871,612	3.57	99.7
CASTLE-2	CGLAB20235385	Tumor	DNA	72,754,092	21.83	99.4
CASTLE-2	CGLAB20246464	Tumor-adjacent	DNA	4,507,196	0.90	99.9
CASTLE-3	CGLAB20235420	Tumor	DNA	65,834,715	19.75	99.6
CASTLE-4	CGLAB20235418	Tumor	DNA	66,523,747	19.96	99.6
CASTLE-4	CGLAB20246472	Tumor-adjacent	DNA	53,579,268	10.72	99.8
CASTLE-5	CGLAB20235419	Tumor	DNA	51,108,390	15.33	99.5
CASTLE-6	CGLAB20235422	Tumor	DNA	65,506,584	19.65	99.5
TSCC-1	CGLAB20235302	Tumor	DNA	64,610,238	19.38	99.5
TSCC-1	CGLAB20246378	Tumor-adjacent	DNA	51,341,687	10.27	99.7
TSCC2	CGLAB20235305	Tumor	DNA	7,984,142	2.40	99.6
TSCC2	CGLAB20246387	Tumor-adjacent	DNA	50,638,948	10.13	99.8
TSCC3	CGLAB20235310	Tumor	DNA	57,584,346	17.28	99.5
TSCC3	CGLAB20246395	Tumor-adjacent	DNA	46,570,554	9.31	99.5
TSCC4	CGLAB20235322	Tumor	DNA	66,693,574	20.01	99.7
TSCC4	CGLAB20246391	Tumor-adjacent	DNA	72,976,478	14.60	99.9
TSCC5	CGLAB20235346	Tumor	DNA	61,889,503	18.57	99.6
TSCC5	CGLAB20246383	Tumor-adjacent	DNA	59,696,443	11.94	99.9
TSCC6	CGLAB20235354	Tumor	DNA	64,957,209	19.49	99.7
TSCC6	CGLAB20246375	Tumor-adjacent	DNA	76,603,027	15.32	99.9
CASTLE-6	CGLAB20235422	Tumor	RNA	96,503,074	28.95	100
TSCC1	CGLAB20235302	Tumor	RNA	109,330,038	21.87	100
TSCC2	CGLAB20235305	Tumor	RNA	65,290,214	19.59	100
TSCC3	CGLAB20235310	Tumor	RNA	103,352,179	31.01	100
TSCC4	CGLAB20235322	Tumor	RNA	75,007,544	22.50	100
TSCC5	CGLAB20235346	Tumor	RNA	92,611,701	27.78	100
TSCC6	CGLAB20235354	Tumor	RNA	78,778,181	23.63	100

Supplementary Table S4. Mutect filtering statistics. The left Sample column indicates diagnosis: CASTLE (orange) and thymic carcinoma (teal).

Patient	Weak evidence	Strand bias	Contamination	Orientation	Slippage	Haplotype	Germline	Matching normal
CASTLE-1	341.8	3.2	131.7	9597.7	0	839.5	32.4	Yes
CASTLE-2	45.4	4.2	6.7	162.6	0	14.4	9.9	Yes
CASTLE-3	276.6	10.8	142.1	3492.2	0	202.2	30	No
CASTLE-4	0.8	0.1	2.8	3.8	0.6	0.1	5	Yes
CASTLE-5	407.1	2.4	16.5	8345.9	0	715.5	48.6	No
CASTLE-6	1.7	0	99.7	37.5	0	2.5	0	No
TSCC-1	142.3	2.3	9.3	2444.6	0	171	0	Yes
TSCC-2	53.1	0.2	0.7	144.4	0	32	0.2	Yes
TSCC-3	293.4	9.6	12.6	2948.8	0	129.6	0	Yes
TSCC-4	130.4	9.7	6.1	429.1	0	16.8	0	Yes
TSCC-5	170.4	9.4	3.8	670.3	0	18.5	0	Yes
TSCC-6	130.9	13.9	2.9	363.4	0	11.7	0	Yes

**Filter tag**	**Description**
Weak evidence	Log-odds score below threshold
Strand bias	Strand bias Phred score
Contamination	Cross-sample contamination
Orientation	OxoG / FFPE read-orientation artifact (C:G→T:A in CCN context)
Slippage	Microsatellite slippage artifact
Haplotype	Variant inconsistent with supporting haplotype
Germline	Evidence of germline variant (using 1000G PON)

Threshold

LOD < --min-allele-fraction 0.05

SB Phred < 0 (flagged by GATK)

Uses per-sample contamination table

Learnt via LearnReadOrientationModel; applied with default prior

--min-slippage-length 8, --pcr-slippage-rate 0.1

Local realignment-based filter

Population AF > 0.01
